# Staging and prognosis of oropharyngeal carcinoma according to the 8th Edition of the American Joint Committee on Cancer Staging Manual in human papillomavirus infection

**DOI:** 10.1007/s00405-018-05263-x

**Published:** 2018-12-29

**Authors:** Yukashi Yamashita, Taro Ikegami, Hitoshi Hirakawa, Takayuki Uehara, Zeyi Deng, Shinya Agena, Jin Uezato, Shunsuke Kondo, Asanori Kiyuna, Hiroyuki Maeda, Mikio Suzuki, Akira Ganaha

**Affiliations:** 1 Department of Otorhinolaryngology, Head and Neck Surgery, Graduate School of Medicine, University of the Ryukyus, Okinawa, 903-0215 Japan; 2 Department of Otorhinolaryngology, Head and Neck Surgery, Zhujiang Hospital, Southern Medical University, Guangzhou, China

**Keywords:** Oropharyngeal cancer, American Joint Committee on Cancer Staging Manual 8th Edition, Human papillomavirus, Viral load, P16 overexpression, Overall survival

## Abstract

**Purpose:**

The aim of this study was to evaluate the 8th edition of the American Joint Committee on Cancer Staging Manual: Head and Neck Section on oropharyngeal squamous cell cancer (OPSCC) and to clarify the relationship between p16 overexpression and the presence of human papillomavirus (HPV) DNA using fresh frozen samples.

**Methods:**

Samples from 100 OPSCC patients were analyzed using polymerase chain reaction (PCR) and p16 immunohistochemistry.

**Results:**

Five-year overall survival (OS) was 73.0%, 93.9%, and 62.2% in all, p16-positive (*n* = 34), and p16-negative (*n* = 66) cases, respectively. OS tended to be better aligned with stage in the 8th edition than in the 7th edition. The 5-year OS was 96.0% in never or light smokers (< 40 pack-years), and 87.5% in heavy smokers (≥ 40 pack-years) in the p16-positive group, respectively (*p* = 0.027). HPV infection was found in 100% of p16-positive and 21.2% of p16-negative cases. The p16-positive cases had higher viral load and integrated physical status than the p16-negative cases. Although 1 case with p16 overexpression showed no PCR amplification using consensus primers, PCR amplification was detected using HPV 16 E6-specific primers.

**Conclusions:**

The 8th edition predicts OPSCC prognosis more accurately than the 7th edition and p16-overexpression is an excellent surrogate marker for detecting HPV infection. Although high-risk-type HPV infection was observed in p16-negative cases, it showed no significant effect in survival outcome.

## Introduction

Human papilloma virus (HPV) infection has become an increasingly common cause of oropharyngeal squamous cell carcinoma (OPSCC) [1, 2]. HPV-mediated OPSCC is fairly responsive to chemoradiotherapy and has a better prognosis than HPV-unrelated OPSCC [3]. Thus, a new staging system was proposed in the 8th edition of the American Joint Committee on Cancer (AJCC) Staging Manual, which reflects HPV infection status in determining the clinical stage of OPSCC [4, 5]. The 8th edition of the AJCC Staging Manual adopted p16 immunohistochemistry findings as a surrogate marker for HPV infection. p16 immunoreactivity is an established surrogate marker for HPV-mediated carcinogenesis in OPSCC in cases where the cutoff point for p16 overexpression is diffuse (≥ 75%) tumor expression, with at least moderate (+ 2/3) staining intensity. A previous report suggested that p16 overexpression might be associated with functional pRb disturbance unrelated to HPV infection [6]. However, a subgroup of OPSCC was found to be p16 positive, had no HPV infection [7], and had worse prognosis compared with p16-positive patients [7, 8]. Although the number is small (3.6%) [7], reliable testing for detecting HPV-mediated OPSCC is important for designing future studies and validating inferences from studies confined to HPV-mediated cancer. In a phase III study from the Trans-Tasman Radiation Oncology Group (TROG 02.02), 13.7% of 102 p16-positive patients were HPV negative [9]. Because the prognosis of HPV-mediated OPSCC is fair, it is possible that even if treatment intensity is lowered, the prognosis will not worsen [10, 11]. These de-escalation studies are fascinating in terms of a paradigm shift in OPSCC therapy, and we also consider that there might be a subgroup without HPV DNA in HPV-mediated OPSCC defined by p16 overexpression.

Comparing the 7th and 8th editions of the AJCC Staging Manual [4, 5] in terms of OPSCC, T categories remain the same with two exceptions: the HPV-mediated OPSCC classification does not include Tis, and the T4b category has been removed from p16-positive OPSCC. There are major changes to the N categories in the 8th edition compared with the 7th edition. cN1 and cN2 are defined as ipsilateral lymph nodes no larger than 6 cm and contralateral or bilateral lymph nodes no larger than 6 cm in HPV-mediated OPSCC. Cases with lymph nodes larger than 6 cm are classified as cN3. By contrast, extranodal extension (ENE) has been added to the cN category in HPV-unrelated OPSCC. While major changes were also made in clinical staging in the 7th versus the 8th edition in HPV-mediated OPSCC, stage IV in particular is reserved for cases with distant metastasis.

The aim of this study was to evaluate the 8th edition in view of survival estimation according to HPV infection status and to clarify the relationship between p16 overexpression and the presence of HPV DNA using fresh frozen samples.

## Patients and methods

### Subjects

This study involved 100 treatment-naïve determined OPSCC patients without distant metastasis who gave written informed consent for study participation. All patients were diagnosed as having OPSCC by pathologic examination of biopsy samples and were treated at the Department of Otorhinolaryngology, Head and Neck Surgery, University of the Ryukyus, between January 2007 and December 2014. Classification of the TNM stage was performed according to both the 7th and 8th AJCC editions [4, 5]. Tissue samples were classified into grades according to the World Health Organization International Histological Classification of Tumors. To determine the clinical stage in multiple primary cancers, patients underwent physical and endoscopic examinations of the upper gastrointestinal tract, ultrasonic inspection of the neck, computed tomography (CT), and ^18^F-fluorodeoxyglucose-positron-emission tomography CT imaging.

This study was approved by the Institutional Review Board of the University of the Ryukyus and was carried out in accordance with the Helsinki Declaration of 1975. Informed consent was obtained from all OPSCC patients before enrollment.

### Treatment

The principal treatment with curative intent for OPSCC was concurrent chemoradiotherapy regardless of the presence of HPV. All patients had CT-assisted three-dimensional radiation treatment planning in the treatment position with mask immobilization. The full treatment protocol was as reported previously [12]. Briefly, the primary lesion and whole neck including bilateral neck lymph nodes were irradiated with 1.8 Gy per day, up to 50.4 Gy. Irradiation to the primary site and metastatic lymph nodes was subsequently boosted with a further 16.2 Gy in nine fractions. Although several chemotherapy regimens were employed, the main chemotherapy regimen was a combination of nedaplatin (CDGP; 90 mg/m^2^ on day 1) and 5-fluorouracil (5-FU, 800 mg/m^2^ on days 2–6). The regimen was given twice with a 4-week interval. When the primary tumor failed to show a partial response regardless of the neck lymph node response at 39.6 Gy irradiation, and patients underwent curative surgery for the primary lesion combined with neck dissection. Also, patients with T1 stage in the 7th edition underwent primary lesion removal with neck dissection in cases where there was no likelihood of severe surgical complications related to swallowing ability and quality of life.

### HPV status and p16 immunohistochemistry

All tissue samples from primary lesions were analyzed with both polymerase chain reaction (PCR) using fresh frozen samples and p16 immunohistochemistry using formalin-fixed paraffin-embedded (FFPE) samples. The detection methods have been published in detail previously [8, 13].

In brief, DNA was isolated from the tumor samples using the Gentra Purification Tissue kit (Qiagen, Germantown, MD). The presence and integrity of the DNA was verified by PCR β-globin gene amplification using the primers PC04 and GH20. The presence of HPV DNA was analyzed with PCR using the consensus primer sets GP5+/GP6 + and MY09/11 [14]. DNA samples that were negative for HPV using GP5+/GP6 + or MY09/11 PCR were re-amplified by auto-nested PCR using the GP5+/GP6 + primer pair. PCR products were purified and directly sequenced with the ABI PRISM 3130 × 1 Genetic Analyzer (Applied Biosystems, Foster City, CA). Obtained sequences were then compared with those of known HPV types in the GenBank database using the BLAST program.

To evaluate the viral load and physical status of HPV-16, quantitative real-time PCR was performed as previously described [13]. Briefly, primers and TaqMan probes targeting the HPV-16 *E2* and *E6* open reading frames were used. The primers and probes recognize the *E2* hinge region, which is deleted on HPV-16 integration. Two standard curves for the *E2* and *E6* genes were created by amplification of tenfold serial dilutions (10^1^, 10^2^, 10^3^, 10^4^, 10^5^, and 10^6^ viral copies) of the plasmid pB-actin carrying the complete HPV-16 early gene region (Addgene plasmid # 13711, Addgene, Cambridge, MA), a gift from Karl Munger. Viral DNA load was assessed by calculating *E6* copy numbers. An external standard curve was created using known serial dilutions (0.3, 3, 30, and 300 ng) of human genomic placental DNA (Sigma-Aldrich; Merck KGaA, Darmstadt, Germany) for cellular DNA quantification and β-globin was amplified as described previously [15]. The amount of DNA was calculated by plotting the Cq values against the logarithm of the standard curve. The physical status of HPV-16 was assessed based on a previously published method [15]. First, the total *E6* copy number in 50 ng cellular DNA was determined. Second, the integrated *E6* was calculated by subtracting the *E2* copy number (episomal) from the total E6 copy number (episomal and integrated). An *E2*/*E6* ratio ≥ 1 indicates the predominance of the episomal form, whereas ratios of *E2* copy number/total *E6* of < 1 indicate the presence of both integrated and episomal forms (mixed form) [13, 15].

For p16 immunohistochemistry, serial 4-µm-thick sections from FFPE samples were deparaffinized in xylene and hydrated in a graded series of alcohol [8]. After epitope retrieval, endogenous peroxidase activity was quenched by incubating the sections in 3% hydrogen peroxide, containing 15 mM sodium azide for 5 min. The sections were subsequently incubated for 30 min at room temperature with a primary monoclonal mouse anti-p16 antibody (MTM Laboratories AG, Heidelberg, Germany). After extensive washing in phosphate-buffered saline, the slides were incubated for 30 min at room temperature with a horseradish peroxidase-conjugated goat anti-mouse secondary antibody (MTM Laboratories). Immunolabeling was visualized by incubation in 3,3′-diaminobenzidine and stained slides were counterstained with hematoxylin. The cutoff point for p16 overexpression was diffuse (≥ 75%) tumor expression, with at least moderate (+ 2/3) staining intensity according to the 8th edition.

It is possible for HPV samples to show negative PCR results when consensus primers are used, particularly in cases where the L1 region is deleted during integration into the host genome. Thus, despite HPV not being detected by PCR using the consensus primer sets GP5+/GP6 + and MY09/11, the samples with p16 overexpression were found to have HPV *E6* and *E2* on further analysis using the above-mentioned method.

### Statistical analysis

Pearson’s chi-square test was used to compare the characteristics of OPSCC patients according to p16 status. Overall survival was calculated using the Kaplan–Meier method and was compared between the two groups using the log-rank test. All analyses were performed with SPSS Statistical Package (SPSS, Version 25.0; SPSS, Inc., Chicago, IL). *p* < 0.05 was considered statistically significant.

## Results

### Characteristics and overall survival of OPSCC patients according to p16 status (Fig. 1)

**Fig. 1:**
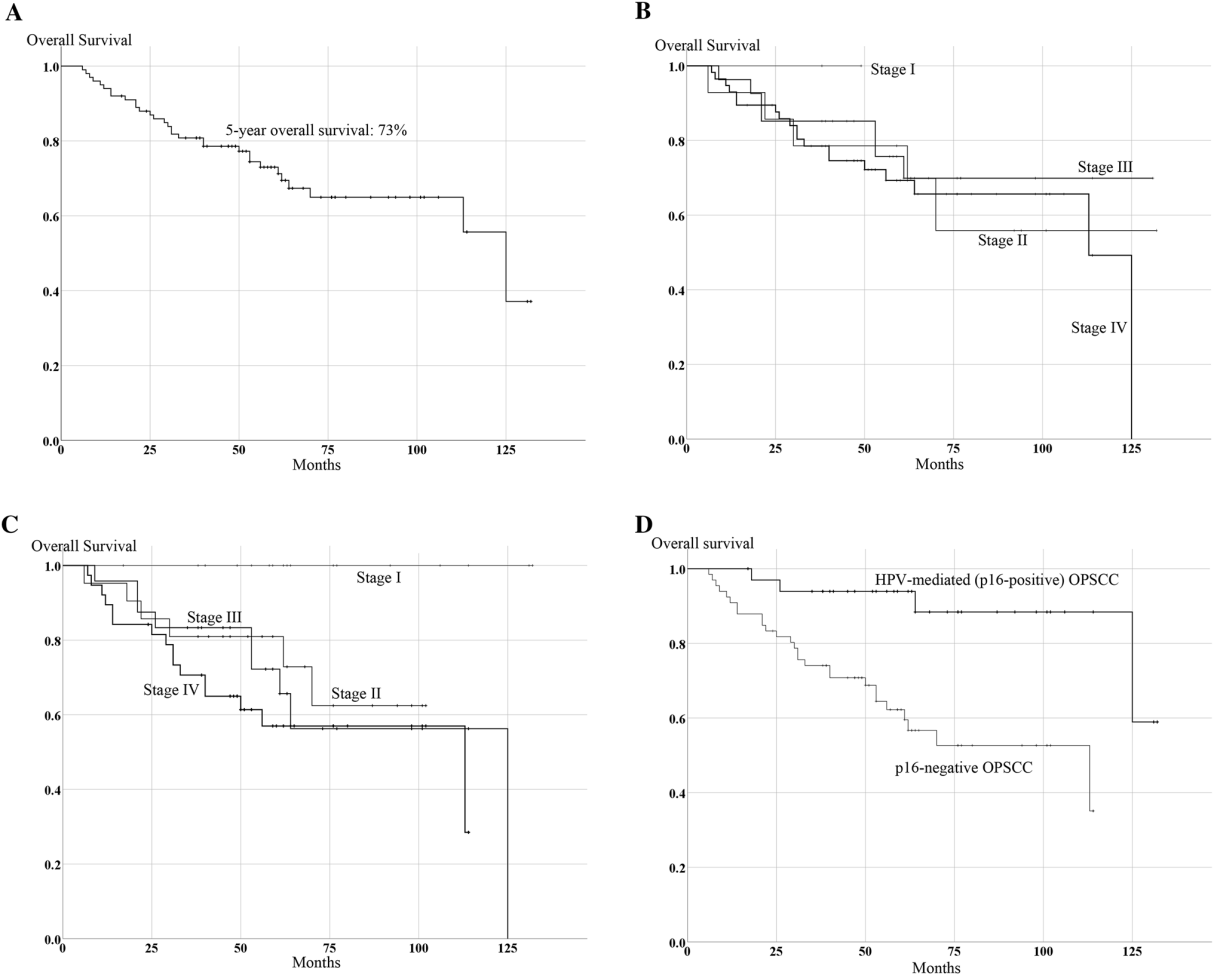
Kaplan–Meier curve of overall survival (OS) in the 100 OPSCC cases. **a** Kaplan–Meier curve of OS in all OPSCC cases. **b** Kaplan–Meier curve of OS according to stages determined by the 7th edition of the AJCC staging manual. **c** Kaplan–Meier curve of OS according to stages determined by the 8th edition of the AJCC staging manual. **d** Kaplan–Meier curve of OS in p16-positive and p-16 negative cases

The clinical characteristics of all OPSCC patients (*n* = 100), p16-positive OPSCC patients (*n* = 34), and p16-negative OPSCC patients (*n* = 66) are shown in Tables 1 and 2. The median follow-up of patients who received treatment and remained alive was 62 months. The 5-year overall survival (OS) was 73.0% in all patients with OPSCC (Fig. 1a), 93.9% in p16-positive patients, and 62.2% in p16-negative patients. There was a significant difference in OS between p16-positive and p16-negative cases (*p* < 0.001, Fig. 1d).

**Table 1:** Characteristics of oropharyngeal squamous cell carcinoma patients according to p16 status

	All cases (*n* = 100)	p16 positive (*n* = 34)	p16 negative (*n* = 66)	*p* value
Sex				0.807
Male	78	27 (79.4)	51 (77.3)	
Female	22	7 (20.6)	15 (22.7)	
Age (years)				0.139
< 63	50	21 (61.8)	29 (43.9)	
≥ 63	50	13 (38.2)	37 (56.1)	
T				0.47
7th and 8th ed T1, T2	47	18 (52.9)	29 (43.9)	
7th and 8th ed T3, T4	53	16 (47.1)	37 (56.1)	
*N*				0.07
7th and 8th ed N0, N1	61	27 (79.4)	34 (51.5)	
7th and 8th ed N2, N3	39	7 (20.6)	32 (48.5)	
SCC differentiation				0.061
Well	28	8 (23.5)	20 (30.3)	
Moderately	57	17 (50.0)	40 (60.6)	
Poorly	12	8 (23.5)	4 (6.1)	
Unknown	3	1 (2.9)	2 (3.0)	
Tumor subsite				0.064
Lateral	63	29 (76.5)	37 (56.1)	
Anterior	26	8 (23.5)	18 (27.3)	
Superior	8	0 (0)	7 (12.1)	
Posterior	3	0 (0)	3 (4.5)	
Second primary				0.013
Number of patients	24	3 (8.8)	21 (31.8)	
Primary treatment				0.029
Surgery ± RT/CCRT CCRT to surgery	26	4 (11.8)	22 (33.3)	
RT or CCRT	74	30 (88.2)	44 (66.7)	
5-Year overall survival (%)	73.0	93.9	62.2	0.001

*CCRT* concurrent chemoradiotherapy, *RT* radiotherapy, *SCC* squamous cell cancer

**Table 2:** Characteristics of OPSCC patients according to p16 status by HPV infection and daily lifestyle habits

	All cases (*n* = 100)	p16 positive (*n* = 34)	p16 negative (*n* = 66)	*p* value
High-risk-type HPV DNA status				< 0.001
Positive	46	34 (100)	14 (21.2)	
Negative	54	0 (0)	52 (78.8)	
HPV types				
HPV 16	38	25	13	
HPV 33	4	3	1	
HPV 35	2	2		
HPV 56	1	1		
HPV 58	2	2		
HPV 67	1	1		
Low-risk-type HPV 6	1		1	
Smoking consumption				0.001
Less than 40 pack-years	53	26 (76.5)	27 (40.1)	
Equal to or more than 40 pack-years	47	8 (23.5)	39 (59.9)	
Alcohol consumption (g/day)				0.003
0–39.9	43	22 (64.7)	21 (31.8)	
≥ 40	57	12 (35.5)	45 (68.2)	

*HPV* human papilloma virus

There were no significant differences in sex, age, T category, N category, SCC differentiation, and tumor subsite between p16-positive and negative cases. There were significant differences in the rate of secondary primary cancer, tobacco consumption, alcohol consumption, and primary treatment between p16-positive and negative cases, as shown in Tables 1 and 2. There was one case with no HPV DNA detected using PCR with consensus primers in the p16-positive group. However, this particular case showed PCR amplification with the use of HPV-16 *E6*-specific primers. The patient in this case had concurrent chemoradiotherapy and survived without recurrence.

**Fig. 2:**
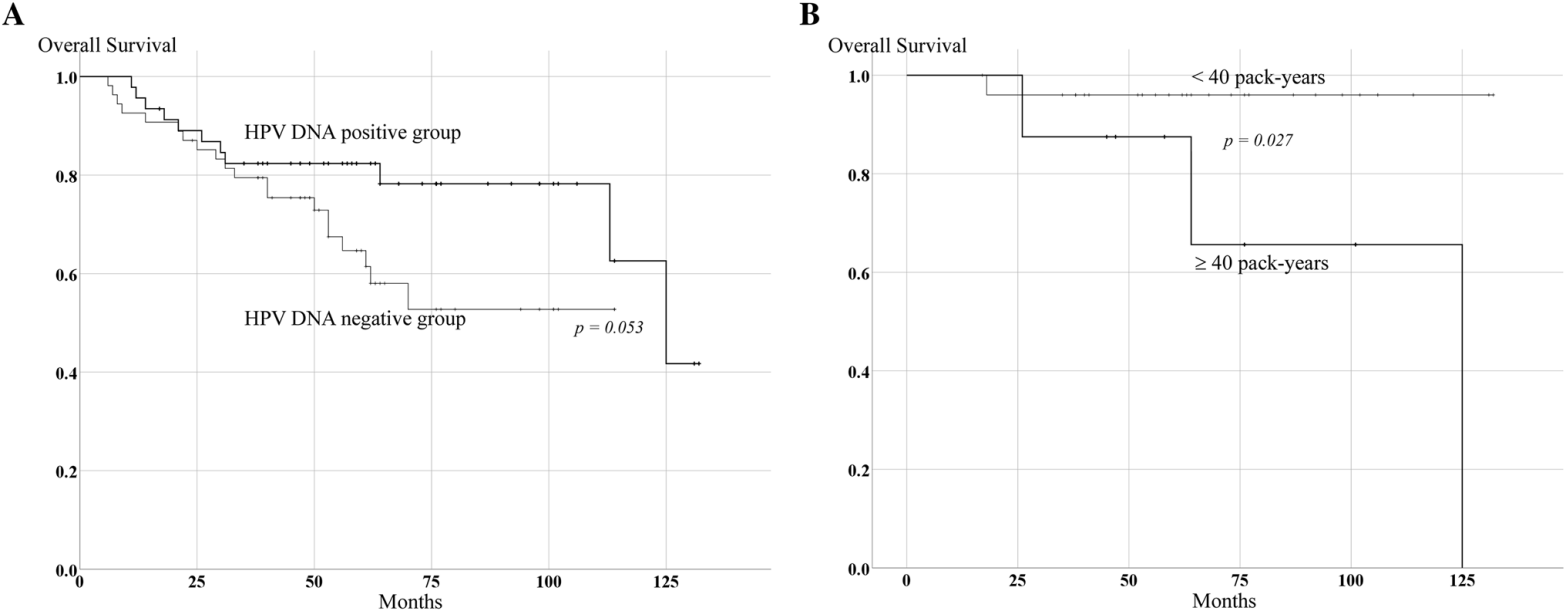
Survival analysis of OPSCC subgroups. **a** Kaplan–Meier curve of OS in HPV DNA-positive and -negative groups. **b** Kaplan–Meier curve of OS by tobacco consumption in HPV-mediated OPSCC

The 5-year OS was 96.0% in never or light smokers (< 40 pack-years), and 87.5% in heavy smokers (≥ 40 pack-years) in the p16-positive group, respectively (*p* = 0.027, Fig. 2b). However, there were no statistical differences in OS related to drinking habit in the p16-positive group (*p* = 0.840) and related to smoking habit and drinking habit in the p16-negative group (*p* = 0.713 and *p* = 0.630, respectively). Of the 100 OPSCC cases, 48.0% were HPV DNA positive from PCR. All p16-positive cases were found to harbor HPV DNA by PCR with consensus primers or HPV 16 *E6*-specific primers; there were also 14 (21.2%) p16-negative cases who harbored HPV DNA. There was substantial agreement between p16 immunohistochemistry and PCR for HPV DNA (kappa = 0.716). According to HPV DNA status, 5-year OS of HPV DNA-positive cases was 83.1%, and for HPV DNA-negative cases was 63.9% in all 100 OPSCC cases. The difference in OS between these two groups was marginal but did not reach significance (*p* = 0.053, Fig. 2a). In the p16-negative group, 5-year OS was 57.1% in HPV DNA-positive cases and 63.9% in HPV DNA-negative cases, respectively (*p* = 0.348).

HPV-16 is the dominant HPV type, comprising 82.6% of all high-risk HPV types. The HPV-16 viral load in 25 cases in the p16-positive group and in 9 cases of p16-negative group could be measured with DNA of a sufficient quality. Marked significant differences in the median value of HPV-16 viral load were observed: 13.33 × 10^5^ copies/50 ng genomic DNA in the p16-positive group and 91 copies/50 ng in the p16-negative group, respectively (*p* < 0.001, Table 3). The physical status in HPV-16 infection could be measured in 25 cases in the p16-positive group and 9 cases in the p16-negative group. Of 25 p16-positive cases, 24 had completely integrated or mixed status, while 55.5% of p16-negative cases had episomal status. The difference between completely integrated or mixed status in both groups reached significance (*p* < 0.001).

**Table 3:** HPV-16 viral load in the p16-positive and p16-negative groups

	All patients (*n* = 100)	p16 positive (*n* = 34)	p16 negative (*n* = 66)
Number of HPV-16 cases	37	25	13
Available samples for viral load	33	25	9
Median		13.33 × 10^5^	91
Minimum		15	12
Maximum		134.67 × 10^5^	98.46 × 10^2^
Available samples for physical status		25	9
Integrated or mixed		24	4
episomal		1	5

*HPV* human papilloma virus

### Staging differences between the 7th and 8th editions of the AJCC staging manual

Staging of the p16-positive OPSCC cases differed considerably between the 7th and 8th editions of the AJCC staging manual (Tables 4, 5). Although the N category in the p16-negative group was changed in the 8th edition, stage distribution in the p16-negative OPSCC did not differ between the 7th and 8th editions (Tables 4, 6). All patients in the p16-positive group showed downstaging (Table 7). In addition, p16-negative cases tended to show N3 lesions in the 8th edition because of ENE (Tables 4, 6).

**Table 4:** Distribution of T and N categories of 100 OPSCC cases according to the 7th edition of the AJCC cancer staging manual

100 cases	N Category				p16 positive	N Category				p16 negative	N Category			
T Category	N0	N1	N2a, b, c	N3	T Category	N0	N1	N2a, b, c	N3	T Category	N0	N1	N2a, b, c	N3
T1	2	1	0	1	T1	0	1	0	0	T1	2	0	0	1
T2	14	12	12	5	T2	5	4	7	1	T2	9	8	5	4
T3	5	9	15	1	T3	1	4	5	0	T3	4	5	10	1
T4a	3	7	8	2	T4a	1	3	1	0	T4a	2	4	7	2
T4b	0	1	2	0	T4b	0	1	0	0	T4b	0	0	2	0

**Table 5:** Distribution of T and N categories in HPV-mediated (p16 positive) cases (*n* = 34) according to the 8th edition of the AJCC cancer staging manual

T Category	N Category			
	N0	N1	N2	N3
T1	0	1	0	0
T2	5	9	2	1
T3	1	6	3	0
T4	1	4	1	0

**Table 6:** Distribution of T and N categories in 66 p16-negative cases according to the 8th edition of the AJCC cancer staging manual

T Category	N Category			
	N0	N1	N2a, b, c	N3a, b
T1	2	0	0	1
T2	9	8	5	4
T3	4	5	6	5
T4a	2	4	5	4
T4b	0	0	2	0

**Table 7:** Stage alteration in HPV-mediated OPSCC in the 7th compared with the 8th edition of the AJCC cancer staging manual

7th Edition	8th Edition	
Stage I	Stage I	0
*n* = 0		
Stage II	Stage I	5
*n* = 5		
Stage III	Stage I	5
*n* = 10	Stage II	5
Stage IV	Stage I	5
*n* = 19	Stage II	7
	Stage III	7

### Overall survival in the 7th and 8th editions of the AJCC staging manual

**Fig. 3:**
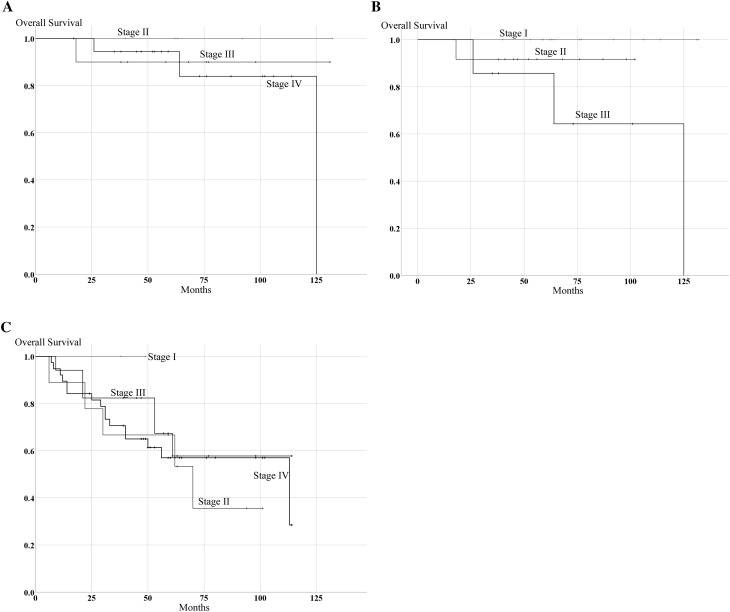
Survival estimation in the p16-positive and p16-negative groups according to the 7th and 8th editions of the AJCC staging manual. **a** Kaplan–Meier curve of OS in HPV-mediated OPSCC according to the 7th edition. **b** Kaplan–Meier curve of OS in HPV-mediated OPSCC according to the 8th edition. **c** Kaplan–Meier curve of OS in the p16-negative OPSCC according to the 7th and 8th editions

Figure 1b, c shows Kaplan–Meier OS curves according to the 7th and 8th editions of the AJCC staging manual in the 100 OPSCC cases. In the 7th edition, 5-year OS for the 100 OPSCC patients was 100% for those with stage I, 78.6% with stage II, 75.7% with stage III, and 69.3% with stage IV, respectively (Fig. 1b). There was no significant difference among stages according to 7th AJCC edition. However, in the 8th edition, 5-year OS for 100 OPSCC patients was 100% for those with stage I, 81.0% with stage II, 72.2% with stage III, and 57.0% with stage IV, respectively (Fig. 1c). Although OS of stages II and III was similar in all 100 OPSCC patients, there were considerable differences between stages I and IV, and between stage I and both stages II and III in the 8th edition. The 5-year OS for p16-positive patients in the 7th and 8th editions is shown in Fig. 3a, b. In the 7th edition, 5-year OS for p16-positive patients was 100% for those with stage II, 90.0% with stage III, and 94.4% with stage IV. In contrast, in the 8th edition, 5-year OS for p16-positive patients was 100% for those with stage I, 91.7% with stage II, and 85.7% with stage IV. There was a significant difference in OS based on the 8th edition in the p16-positive group (*p* = 0.05). Since clinical stages in the p16-negative group were identical in both the 7th and 8th editions, there were no significant differences in OS in the p16-negative group based on both editions (Fig. 3c).

## Discussion

There have been contradictory reports regarding p16 overexpression and HPV status. Possible reasons for such discrepancy among studies include the definition of p16 overexpression, detection methods for HPV infection (in situ hybridization, PCR), and type of sample preparations used for analysis (fresh frozen or FFPE samples) [8, 9, 16–18]. In the present study, based on the use of fresh frozen samples and consensus primers for PCR, 100% of subjects in the p16-positive group had HPV DNA with a high viral load. On the contrary, although 21.2% of the p16-negative cases also had HPV DNA, the viral load was markedly lower than in p16-positive cases. These results suggest that high-risk HPV infection occurs in OPSCC with or without viral transcriptional activity. Our previous study showed that p16 overexpression adequately corresponds to *E6*/*E7* mRNA expression in OPSCC, but not in other head and neck cancers [8]. Because the HPV-16 viral load in OPSCC was much higher than in other head and neck lesions, the high viral load may be related to induction of p16 upregulation through the E6/E7 oncoprotein in OPSCC. In another study that used a PCR protocol similar to ours except for FFPE samples, 85.7% of p16-positive cases harbored HPV DNA and all p16-negative cases were HPV DNA negative [19]. Contrarily, another report demonstrated that only 28% of HPV positive cases showed p16 overexpression [20]. A recent study reported that the sensitivity of droplet digital PCR (ddPCR) of oropharyngeal swabs analyzed for *E6*/*E7* mRNA expression was 92% for p16 overexpression [21]. Furthermore, 1 of 34 ddPCR-positive cases was p16 negative, and 3 of 18 negative ddPCR cases were p16-positive. In our series, of 46 HPV DNA-positive cases, 34 (73.9%) were p16 positive. The PCR method for the detection of *E6*/*E7* mRNA expression [21] might be beneficial in evaluating viral transcriptional activity and carcinogenic involvement of HPV than a PCR method that utilizes L1 consensus primers as in the present study. According to our previous report, the HPV-16 viral load in OPSCC was closely related to *E6*/*E7* mRNA expression [8, 13]. In the present study, however, because cases with high HPV-16 viral load exclusively showed p16 overexpression, p16-positivity criteria defined by the 8th edition surely indicated HPV infection with transcriptional activity in OPSCC as a surrogate marker. Immunohistochemical staining for p16 is inexpensive and is universally available for retrospective examination. These results suggest that p16 immunostaining is cost effective and reliable for detecting HPV-mediated OPSCC.

The p16-positivity criteria in the 8th edition states that the cutoff point for p16 overexpression is diffuse (≥ 75%) tumor expression with at least moderate (+ 2/3) staining intensity [5]. The present study clearly showed that p16-positive cases had a much better prognosis than p16-negative cases. In addition, HPV DNA status had less impact on OS than p16-overexpression. Since HPV DNA-positive cases in the p16-negative group usually had a small viral load, the detected HPV infection may not be pathologic and may exist as a bystander without any viral transcriptional activity of oncoprotein. However, half of these cases showed mixed or complete integration, and thus HPV might still possibly exert some influence on carcinogenesis in OPSCC. This point should be investigated in the near future.

Interestingly, 1 out of the 100 OPSCC cases had no HPV DNA on PCR using consensus primers with p16 overexpression. Previous studies with a large sample size showed that approximately 3–4% of OPSCC had p16 overexpression without HPV infection [7, 22]. They reported that patients with p16 positive but HPV DNA-negative tumors showed significantly less favorable survival than patients with p16-positive and HPV DNA-positive tumors. In the present study, the p16-positive case was proven to have HPV infection on PCR using HPV 16 *E6-*specific primers. Although the number of p16-positive cases without HPV DNA was limited, the cause of p16 overexpression and survival outcome of these cases should be clarified in the near future to aid the design of a clinical trial.

Several studies have indicated that the 7th edition of the AJCC staging system has not properly described prognosis in HPV-mediated OPSCC [3, 23]. The efficacy of the 8th edition on survival estimation was investigated in the present study. In HPV-mediated OPSCC, the N classification was changed in the 8th edition compared with the 7th edition [5]. In the present study, majority of patients with HPV-mediated OPSCC who were N2b in the 7th edition became N1 in the 8th edition. Therefore, patients in stages III and IV in the 7th edition became stage I or II in the 8th edition. There was no significant difference in OS among stages in the 7th edition in the present study. However, in the 8th edition, OS rates in stages I, II, and III were aligned according to each stage. Similar findings were obtained in p16-negative OPSCC. Stage I p16-negative cases showed better prognosis than other stages, and stage IV cases had the worst prognosis among all stages. These results reveal that the 8th edition is more appropriate for evaluating treatment outcome than the 7th edition. Also, smoking status in HPV-mediated OPSCC was related to OS and this is consistent with previous reports [3, 23, 24]. Smoking status could be included in the inclusion criteria of a future clinical study. Taken together, these findings are also important for patient inclusion criteria in a clinical study.

In conclusion, the 8th edition of the AJCC staging manual reflects the prognosis of OPSCC using p16 immunoreactivity for the detection of HPV-mediated OPSCC. However, a further study is needed to clarify the nature of cases that are p16 negative with HPV infection. The discordance between p16 overexpression and the presence of HPV DNA in relation to survival outcome should be clarified to design a de-escalation study.

## References

[1] Nasman A, Attner P, Hammarstedt L (2009). Incidence of human papillomavirus (HPV) positive tonsillar carcinoma in Stockholm, Sweden: an epidemic of viral-induced carcinoma?. Int J Cancer.

[2] Chenevert J, Chiosea S (2012). Incidence of human papillomavirus in oropharyngeal squamous cell carcinomas: now and 50years ago. Hum Pathol.

[3] Ang KK, Harris J, Wheeler R (2010). Human papillomavirus and survival of patients with oropharyngeal cancer. N Engl J Med.

[4] O’Sullivan B, Huang SH, Su J (2016). Development and validation of a staging system for HPV-related oropharyngeal cancer by the International Collaboration on Oropharyngeal cancer Network for Staging (ICON-S): a multicentre cohort study. Lancet Oncol.

[5] Doescher J, Veit JA, Hoffmann TK (2017). The 8th edition of the AJCC Cancer Staging Manual: updates in otorhinolaryngology, head and neck surgery. HNO.

[6] Marur S, D’Souza G, Westra WH, Forastiere AA (2010). HPV-associated head and neck cancer: a virus-related cancer epidemic. Lancet Oncol.

[7] Rietbergen MM, Brakenhoff RH, Bloemena E (2013). Human papillomavirus detection and comorbidity: critical issues in selection of patients with oropharyngeal cancer for treatment De-escalation trials. Ann Oncol.

[8] Deng Z, Hasegawa M, Aoki K (2014). A comprehensive evaluation of human papillomavirus positive status and p16INK4a overexpression as a prognostic biomarker in head and neck squamous cell carcinoma. Int J Oncol.

[9] Rischin D, Young RJ, Fisher R (2010). Prognostic significance of p16INK4A and human papillomavirus in patients with oropharyngeal cancer treated on TROG 02.02 phase III trial. J Clin Oncol.

[10] Dronkers EAC, Koljenovic S, Verduijn GM, Baatenburg de Jong RJ, Hardillo JAU (2018). Nodal response after 46Gy of intensity-modulated radiotherapy is associated with human papillomavirus-related oropharyngeal carcinoma. Laryngoscope.

[11] Stock GT, Bonadio R, de Castro GJ (2018). De-escalation treatment of human papillomavirus-positive oropharyngeal squamous cell carcinoma: an evidence-based review for the locally advanced disease. Curr Opin Oncol.

[12] Hasegawa M, Maeda H, Deng Z (2014). Prediction of concurrent chemoradiotherapy outcome in advanced oropharyngeal cancer. Int J Oncol.

[13] Deng Z, Hasegawa M, Kiyuna A (2013). Viral load, physical status, and E6/E7 mRNA expression of human papillomavirus in head and neck squamous cell carcinoma. Head Neck.

[14] Deng Z, Hasegawa M, Matayoshi S (2011). Prevalence and clinical features of human papillomavirus in head and neck squamous cell carcinoma in Okinawa, southern Japan. Eur Arch Otorhinolaryngol.

[15] Peitsaro P, Johansson B, Syrjanen S (2002). Integrated human papillomavirus type 16 is frequently found in cervical cancer precursors as demonstrated by a novel quantitative real-time PCR technique. J Clin Microbiol.

[16] Bussu F, Sali M, Gallus R (2013). HPV infection in squamous cell carcinomas arising from different mucosal sites of the head and neck region. Is p16 immunohistochemistry a reliable surrogate marker?. Br J Cancer.

[17] Gronhoj Larsen C, Gyldenlove M, Jensen DH (2014). Correlation between human papillomavirus and p16 overexpression in oropharyngeal tumours: a systematic review. Br J Cancer.

[18] Melkane AE, Mirghani H, Auperin A (2014). HPV-related oropharyngeal squamous cell carcinomas: a comparison between three diagnostic approaches. Am J Otolaryngol.

[19] Dreyer JH, Hauck F, Oliveira-Silva M, Barros MH, Niedobitek G (2013). Detection of HPV infection in head and neck squamous cell carcinoma: a practical proposal. Virchows Arch.

[20] Orsaria M, Marzinotto S, de marchi L (2015). HPV-related oropharyngeal squamous cell carcinoma: p16I^NK4A^ immunohistochemistry or HPV genotyping?. Anticancer Res.

[21] Isaac A, Kostiuk M, Zhang H (2017). Ultrasensitive detection of oncogenic human papillomavirus in oropharyngeal tissue swabs. J Otolaryngol Head Neck Surg.

[22] Mena M, Taberna M, Tous S (2018). Double positivity for HPV-DNA/p16(ink4a) is the biomarker with strongest diagnostic accuracy and prognostic value for human papillomavirus related oropharyngeal cancer patients. Oral Oncol.

[23] Gillison ML, D’Souza G, Westra W (2008). Distinct risk factor profiles for human papillomavirus type 16-positive and human papillomavirus type 16-negative head and neck cancers. J Natl Cancer Inst.

[24] Hooper JE, Hebert JF, Schilling A (2015). Hybrid capture 2 is as effective as PCR testing for high-risk human papillomavirus in head and neck cancers. Appl Immunohistochem Mol Morphol.

